# Preventive behaviour and attitudes towards early childhood caries amongst mothers of toddlers in Bangladesh

**DOI:** 10.1080/00016357.2023.2291205

**Published:** 2024-03-26

**Authors:** Farzana Haque, Morenike O. Folayan, Jorma I. Virtanen

**Affiliations:** a Department of Clinical Dentistry, University of Bergen, Bergen, Norway; b Department of Child Dental Health, Obafemi Awolowo University, Ile-Ife, Nigeria; c Institute of Dentistry, University of Turku, Turku, Finland

**Keywords:** Early childhood caries, children, mothers, prevention, attitudes

## Abstract

**Background:**

Our aim was to analyse mothers of toddlers’ preventive behaviour towards ECC in Bangladesh.

**Methods:**

We conducted a cross-sectional survey of mothers and their 6–24-month-old children visiting vaccination centres in Trishal, Bangladesh in 2021. A cluster-sampling method was applied to select 10 immunization centres and all mothers who attended the centres with a 6–24-month-old child were recruited. Mothers’ preventive behaviour and attitude towards oral health was determined using a reliable instrument. Clinical examinations were conducted to assess the presence of dental plaque on the labial surfaces of the upper central incisors and the ICDAS II index criteria were utilized to detect ECC. The associations between preventive behaviours and the plaque score and caries status of the children were determined using multivariable logistic regression analysis after adjusting for confounding variables (mother’s age and educational status).

**Results:**

The prevalence of ECC among the children was 25.8%. ECC experience was significantly associated with low educational level (*p* = 0.02) and older age (*p* = 0.01) of mothers. Of the mothers, 75.2% reported to brush their teeth twice daily and about half of them (48.8%) cleaned their children’s teeth daily; and 5.8% with fluoridated toothpaste. The multivariate logistic regression analysis showed that caries preventive behaviour of mothers (AOR = 2.63, 95% CI 1.41–4.91) and the plaque score of the child (AOR = 14.69, 95% CI 7.45–28.9) were significant risk indicators for ECC in the study population.

**Conclusions:**

The prevalence of ECC was high among the Bangladeshi toddlers and factors such as the mothers’ preventive behaviour and presence of plaque were associated with the occurrence.

## Introduction

Early Childhood Caries (ECC) is a prevalent chronic condition affecting 530 million children worldwide in 2019, making it one of the most common diseases among children [1]. ECC refers to tooth decay on any surface of primary teeth and is typically observed in the first three years of life in Europe and within the first six years in the USA [2,3]. If left untreated, ECC can progress to a more severe form of the disease, resulting in issues such as malocclusions, abscess pain [4], poor oral health-related quality of life, malnutrition, and growth deficits [4,5].

The prevalence of ECC is influenced by national healthcare systems and access to preventive and curative care [6]. However, data on ECC is often limited at both the country and global levels [6]. While poverty is associated with ECC, countries with higher gross national product (GNP) may also experience higher ECC rates [6,7]. Moreover, ECC prevalence tends to be higher in countries with a larger percentage of the population living in slums or below the poverty line [7].

In Bangladesh, there is great demand for greater awareness of oral health and prevention of dental caries [8]. The country has limited dental services, poor ratio of dental healthcare providers to the population is high (100,000:6) [9] and a high prevalence of dental caries among children 2- to 5-year-old children [8,10]. Maternal knowledge and practices related to ECC development is low, suggesting a lack of awareness among parents regarding the importance of oral care for their children [11]. Global evidence suggests that not only do maternal factors such as low education level, increase the risk of preschool children to ECC, also children’s high sugar intake, poor oral hygiene, and limited fluoride exposure contribute to this risk [12–15]. Maternal education level is associated with oral health habits in children, such as inadequate tooth brushing, high sugar consumption, and consuming refined carbohydrates before bedtime [15,16]. It is, therefore, important, to identify the mix of sociodemographic and behavioural risk factors that predisposes children in a context like Bangladesh, to ECC to be able to design and implement effective ECC control strategies.

This study is grounded in the theory of planned behaviour [17], which emphasizes the role of intention in driving behaviour. According to this theory, an individual’s intention to engage in a specific behaviour is shaped by their attitudes, subjective norms, and perceived behavioural control. These factors collectively influence the strength of the intention to exhibit a particular behaviour. By understanding the factors that influence individual behaviour, effective strategies can be developed to address these factors and increase the likelihood of positive behaviour change.

This study aims to investigate the preventive behaviours and attitudes towards oral health among mothers of toddlers aged 6-24 months in Trishal, Bangladesh. Additionally, the study aims to identify risk indicators and preventive strategies for ECC in infants. The hypothesis is that poor caries preventive behaviours among mothers is associated with an increased risk of ECC.

## Methods

Ethical approval for the study (MMC/RB/2021/349) was granted by the Institutional Review Board at Mymensingh Medical College, with additional approval from the Mymensingh civil surgeon office. The study adhered to the guidelines of the Declaration of Helsinki. We informed the parents/guardians that the information of the study is anonymous and will be beneficial for future for children who are prone to develop ECC. The study was voluntary and all requested information was completely confidential. A written informed consent was obtained from the mothers prior to participation.

### Study design and study site

This cross-sectional clinical study was conducted in Trishal, Bangladesh, in 2021. Trishal is a middle-income town in Mymensingh district with a population of approximately 480,196. People in Trishal are aware of inoculation program offered to all children despite the low literacy rate [18]. The coverage of the vaccinations in Trishal is high, about 98% of all children participate in the program. In addition to the child vaccinations, counselling of the parents regarding immunization and other diseases like diarrhoea, common cold etc. is provided. Dental care is available at the Upazila health complex hospital for urgent care and in private clinics. In Mymensingh the fluoride content in water ranges between 0.04 and 0.63 mg/L [19].

### Study population

The study recruited mother-child dyads who visited the Extended Program of Immunization (EPI) vaccination centres in Trishal between August and October 2021. Target subjects included mothers with a child between 6–24-months visiting the EPI vaccination centres in Trishal. The children without any teeth were excluded from the study.

### Sampling procedure

A cluster sampling method was utilized to select the EPI centres from the list provided by Trishal Upazila health complex. Ten centres were randomly selected from the 40 big centres with at least 20 mother-child visits per day. All mothers who were willing to participate were invited to the study.

Data was collected through an interviewer administered questionnaire. A trained dentist who is fluent in the local language and familiar with local norms (FH) collected the data from study participants with the help of vaccination assistants. The mothers were interviewed after they had completed the child’s vaccination schedules. The study questionnaire was first administered and then a clinical examination for the child was conducted. The survey and clinical examinations were conducted using previously used method [14,15,20].

### Questionnaire

To gather data from the mothers, a validated questionnaire [20–22] was employed. The questionnaire was originally in English and was subsequently translated into Bangla and back translated by an independent bilingual translator. It consisted of three sections: the background section, which focused on the mother’s socio-economic characteristics and tooth cleaning practices; the second section, which assessed the mother’s attitude towards oral health; and the third section, which inquired about the use of dental services and ECC preventive behaviour. The questionnaire underwent three iterative processes for content validation, and the Cronbach’s alpha of the tool was 0.78.

The section on preventive behaviour had four questions that enquired about the use of fluoridated toothpaste, frequency of tooth brushing, frequency of intake of sugary snacks, and frequency of adding sugar to the feeding bottle content. The response alternatives were ‘yes’ or ‘no’. The responses were scored as 0 for ‘Yes’ and 1 for ‘No’. The sum of these scores served as the caries preventive behaviour score for each respondent.

In the section on the use of dental services, two questions asked about visiting the dentist for regular dental check-up and dental examination by any health care provider. The response options for both questions were ‘yes’ or ‘no’. The responses were scored as 0 for ‘Yes’ and 1 for ‘No’. The sum of these scores represented the healthcare score for each respondent.

The section on the mother’s attitude included nine statements about parents’ opinions, intentions, and perceptions regarding their child’s tooth brushing and daily consumption of sugary products. The responses were on a 5-point Likert scale ranging from ‘Strongly Agree’ to ‘Strongly Disagree’. The responses were scored from 1 to 5, with 1 and 2 indicating agreements, and 4 and 5 indicating disagreements. The sum of these scores represented the final oral health attitude score for each mother.

The background information collected include the following: mother’s age (years), educational level (basic, primary, secondary, and tertiary education), number of children in the family, child’s age (months), and tooth brushing habits (twice a day, once a day, and sometimes/never).

For the multivariate analysis, the sum scores for caries preventive behaviour, use of dental services, and attitude were dichotomized. Mother’s caries preventive behaviour was categorized as either poor (0–2) or good (3–4), use of dental services as poor (0) or good (1–2), and attitude as poor (0–6) or good (7–9). Plaque scores were categorized as 0 for no plaque and 1 for the presence of plaque on any tooth surface. ECC was categorized as 0 for no caries (ICDAS = 0) and 1 for the presence of caries (ICDAS = 1–6) in one or more teeth. Mother’s age was categorized as younger mother (<25 years) and older mother (≥25 years), and their education level was categorized as high (secondary/tertiary), or low (basic/primary).

### Clinical examinations

Children were clinically examined with the mother and the examiner (FH) sitting in a knee-to-knee position in an examination room [14,20]. The examinations were carried out by lifting child’s lip to check for presence of plaque and early signs of tooth decay with the help of a headlamp, a WHO CPI probe, and a plane dental mirror. The occurrence of any sign of dental caries on any tooth surface as defined by the American Academy of Pediatric Dentistry was classified as ECC [2]. We used the ICDAS II index criteria (ranging from 0 to 6) for the clinical examinations [23]. ECC was categorized as 0 for no caries (ICDAS = 0) and 1 for the presence of caries (ICDAS = 1–6) in one or more teeth.

Visible dental plaque was assessed on the labial surfaces of the upper central incisors and recorded as ‘No visible plaque’, ‘Plaque present at gingival margin only’, and ‘Abundant dental plaque covering more than gingival margin of the tooth’ [14,20].

### Standardization of examiner

Prior to the study, the examiner received additional training from an experienced dentist who is the head of a university department of paediatrics (JV). The pilot examinations for 30 children were conducted within two weeks interval. The Kappa-values for the clinical caries diagnoses in the test-retest analysis of was 0.933 and for plaque 0.576.

### Statistical analyses

IBM SPSS Statistics version 23.0 was used to perform the statistical analyses. The chi-square test was used to assess associations between mother’s socio-economic characteristics and ECC. A multivariate regression model was constructed to determine the independent variable (mothers’ attitude towards oral health, child’s preventive behaviour and use of dental services) associated with the presence of ECC after adjusting for confounders (maternal age, maternal educational level, number of children in the family, child’s age, and tooth brushing habits). Adjusted odds ratios (AOR), 95% confidence intervals (CI), and p-values were calculated, with statistical significance set at 0.05.

## Results

A total of 330 mother-child pairs participated in the study. The characteristics of the participants is presented in Table 1. The mean age of the mothers was 25.1 (SD = 4.8) years. In addition, 148 (44.8%) were 24 years or younger, 55 (16.7%) had tertiary education, and 248 (75.2%) reported brushing their teeth twice daily. There were 151 (45.8%) children within 12 months of age.

**Table 1:** Basic characteristics of the participants (*n* = 330) from Trishal, Bangladesh.

Characteristics	Values	n (%)	Total n (%)
Age of mother (years)	17–24	148 (44.8)	330 (100)
	25–30	148 (44.8)	
	>30	34 (10.3)	
Age of child (months)	6–12	151 (45.8)	330 (100)
	13–18	132 (40.0)	
	19–24	47 (14.2)	
Mother’s education	Basic	27 (8.2)	330 (100)
	Primary	104 (31.5)	
	Secondary	144 (43.6)	
	Tertiary	55 (16.7)	
Mothers’ tooth brushing	Twice daily	248 (75.2)	330 (100)
	Once daily	82 (24.8)	
	Sometimes or never	0	

The mother’s preventive behaviour is summarized in Table 2. Of the children, 6 (1.8%) had visited the dentist during the last year. Of the mothers, 97 (29.4%) reported brushing their children’s teeth twice daily, 49 (14.8%) added sugar to feeding bottle content and 164 (49.7%) give their children sweet or sugary foods once a day or more.

**Table 2:** Mothers’ (*n* = 330) caries preventive behaviours and use of dental services.

Variable	Yes	No	Total
	n (%)	n (%)	n (%)
**Mothers’ caries preventive behaviour**			330 (100)
When your child’s teeth are brushed, is fluoridated toothpaste usually used? (yes)	67 (20.3)	263 (79.7)	
Do you add sugar to milk/water in the bottle?	49 (14.8)	281 (85.2)	
Do you brush your child’s teeth at least once a day?	161 (48.8)	169 (41.2)	
Do you give your child sweet or sugary snacks once a day or more?	164 (49.7)	166 (50.3)	
**Children’s use of dental services**			330 (100)
During the past year, has your child been to the dentist or dental clinic for a routine check-up or cleaning? (yes)	6 (1.8)	324 (98.2)	
Has your child ever had his/her teeth checked by a dentist or other care provider? (yes)	9 (2.7)	321 (97.3)	

**Figure 1:**
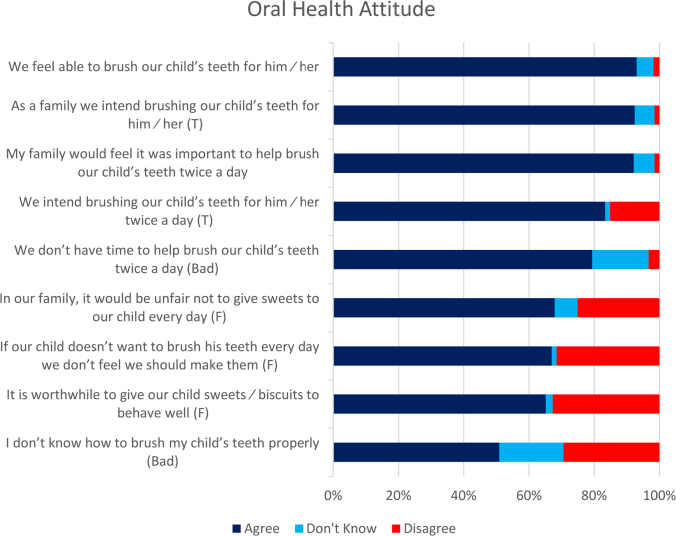
Mothers’ (*n* = 330) oral health attitude.

The mothers’ attitudes towards their children’s oral health are illustrated in Figure 1. Most of the mothers (305, 92.4%) had positive attitude towards tooth brushing of child’s teeth and strongly agreed of the importance of twice daily tooth brushing. Of the mother, 55 (16.7%) mothers, reported not having time to help brush their child’s teeth, however 162 (49.1%) reported not to know how to brush their child’s teeth.

The clinical examinations revealed that the prevalence of ECC among the children was 25.8%. (Table 3). The prevalence of ECC was significantly associated with mother’s age (*p* = 0.01); more children of younger mothers’ children were free of ECC compared to the older mothers’ children. The prevalence of ECC was also significantly associated with mother’s level of education (*p* = 0.02); more children of mothers with higher educational level were free of ECC.

**Table 3:** Background characteristics of the Bangladeshi 6-24-month-olds in Trishal, Bangladesh (*n* = 330) and ECC.

Characteristics	Value	No ECC	ECC present	p-value
Age of mother (years)	Younger mother (<25 years)	120 (81.1)	28 (18.9)	0.01
	Older mother (≥25 years)	125 (68.7)	57 (31.3)	
Mother’s education	High education (Secondary/Tertiary)	156 (78.9)	42 (21.1)	0.02
	Low education (Basic/Primary)	89 (68.7)	43 (31.3)	

Table 4 presents the results of the multivariate logistic regression analysis of the risk indicators for ECC. Caries preventive behaviour of the mothers (AOR = 2.63, 95% CI 1.41– 4.91) and the plaque score in the children (AOR = 14.69, 95% CI 7.45–28.9) were significantly associated with ECC in the study population.

**Table 4:** Factors associated with ECC among the 6-24-months-olds (*n* = 330) in Trishal, Bangladesh explained by logistic regression model.

Variables	Total *N* = 532 (%)	ECC		AOR; 95% CI; *p*-value
		No Caries *N* = 245 (74.2) *n* (%)	Caries present *N* = 85 (25.8) *n* (%)	
**Caries preventive behaviour**				2.63; 1.41-4.91; 0.002
Good Behaviour (ref)	177 (53.6)	142 (80.2)	35 (19.8)	
Poor Behaviour	153 (46.4)	103 (67.3)	50 (32.7)	
**Use of dental services**				0.28; 0.06-1.22; 0.091
Good Service (ref)	12 (3.6)	8 (66.7)	4 (33.3)	
Poor Service	318 (96.4)	237 (74.5)	81 (25.5)	
**Attitude Score**				0.89; 0.48-1.65; 0.724
Good Attitude (ref)	212 (64.4)	161 (75.9)	51 (24.1)	
Poor Attitude	117 (78.8)	84 (28.2)	33 (67.3)	
**Plaque score**				14.69; 7.45-28.9; <0.001
No plaque (ref)	244 (73.9)	209 (75.7)	35 (14.3)	
Plaque present	86 (26.1)	36 (41.9)	50 (58.1)	

Adjusted for: mother’s age, and educational status.

Omnibus test of model coefficients Chi-square = 91.09; *p* < 0.001.

Nagelkerke R2 0.354.

## Discussion

The study showed that prevalence of ECC was high among children 6–24-month-olds with low socioeconomic status in the Bangladeshi: one in four toddlers had ECC. In addition, prevalence of ECC was likely to be higher in children with mothers who had poor preventive oral health behaviour, and in children with plaque in the mouth. These findings support the study hypothesis that poor caries preventive behaviours among mothers is associated with an increased risk of ECC.

To our knowledge, this is the first study about ECC among toddlers in Bangladesh. The use of ICDAS criteria for the diagnosis of ECC increases the validity of the prevalence of ECC for the study population. ICDAS is recommended for the diagnosis of ECC [23]. Radiographic evaluation could have, however, improved the validity of the results. Another study limitation is the focus on mothers with under 2-year-olds, from one region in Bangladesh and with low socio-economic status thus, the study cannot be generalized to preschool children in the country. In addition, this was a cross-sectional study and thus, a cause-inference deduction cannot be made. Despite these limitations, the study highlights a few important information.

The high prevalence of ECC among 6- to 24-month-old toddlers is in line with the report from India (27.5% and 35.44%, respectively) [13,24] and Sri Lanka (32.2%) [25], but lower than the findings from Thailand (58.4%) [26]. Contrary to our findings, much lower percentage has been reported from Europe (2.9%) [27]. The observed high prevalence of ECC in Bangladesh is attributable to the observed infant feeding practice: in our study about half of the children consumed sugary snacks or food once a day or more. Similar findings have been observed in other countries too [28]. High quantity and frequency of consumption of refined carbohydrate had been associated with ECC experience [15]. Additionally, the observed high plaque scores often resulting from poor plaque control is a known risk factor for ECC also [29].

Inadequate plaque control and high sugar consumption have been found to be associated with poor maternal oral health behaviours [1]. Often, these oral health behaviours are linked to low socioeconomic status, which may result in limited access to information [7]. This study focused on a homogeneous population of households with low socioeconomic status, yet distinctive features of Early Childhood Caries (ECC) were observed, such as poor maternal behaviour and plaque control. The findings suggest that factors other than socioeconomic status may be driving poor maternal behaviour. A previous study indicates that women perceive health protection as good nutrition and regular preventive examinations [30]. However, there is limited knowledge about how mothers perceive oral health for their children and the factors influencing their behaviours towards implementing ECC preventive care for their children. Further investigation is needed to explore this aspect.

In addition, a significant proximal risk factor for ECC is maternal behaviour towards oral health [28,31]. The provision of oral health information can play a crucial role in enhancing positive oral health behaviour and caries preventive practices [32]. In our study, we found that although nearly two-thirds of the mothers had positive attitudes towards maintaining dental hygiene, approximately half of them lacked awareness of the correct technique for brushing their child’s teeth. These findings align with a previous study on ECC risk factors conducted in Bangladesh [10] and are consistent with reports from other low and middle-income countries [33,34]. These findings suggest that access of mothers to oral health education may improve their oral health behaviour and ECC preventive practices. Practices that facilitate mothers’ access to information during the antenatal and postnatal care periods have been effective in improving oral hygiene practices [35].

We acknowledge the limitations of our study, as we understand that ECC is a complex disease influenced by multiple etiological factors at the micro, meso, and macro levels. While our findings and recommendations can potentially contribute to actions at the micro- and meso-levels, they may not directly lead to significant changes in ECC prevalence at the country level. It is important to note that this study represents one of the few investigations on ECC concerning children in Bangladesh. To bring about meaningful change and address the rising prevalence of ECC in the country, further robust research is warranted. Additional studies are needed to challenge the existing paradigm and provide a more comprehensive understanding of ECC in Bangladesh.

In conclusion, the prevalence of ECC is high among toddlers in Bangladesh with low socioeconomic status. Children whose mother had poor ECC preventive behaviour and children with high plaque scores seem more likely to have ECC. Efforts need to be invested in improving maternal access to oral health preventive oral health care in ways to transform their behaviour.
